# Getting miRNA Therapeutics into the Target Cells for Neurodegenerative Diseases: A Mini-Review

**DOI:** 10.3389/fnmol.2016.00129

**Published:** 2016-11-22

**Authors:** Ming Ming Wen

**Affiliations:** Department of Pharmaceutics, Pharos University in AlexandriaAlexandria, Egypt

**Keywords:** miRNA, anti-miR, RNAi, gene, molecular therapeutics, neurodegenerative disease

## Abstract

miRNAs play important roles in modulating gene expression in varying cellular processes and disease pathogenesis, including neurodegenerative diseases. Several miRNAs are expressed in the brain, control brain development and are identified as important biomarkers in the pathogenesis of motor—and neuro-cognitive diseases such as Alzheimer’s (AD), Huntington’s and Parkinson’s diseases (PD) and amyotrophic lateral sclerosis. These remarkable miRNAs could be used as diagnostic markers and therapeutic targeting potential for many stressful and untreatable progressive neurodegenerative diseases. To modulate these miRNA activities, there are currently two strategies involved; first one is to therapeutically restore the suppressed miRNA level by miRNA mimics (agonist), and the other one is to inhibit miRNA function by using anti-miR (antagonist) to repress overactive miRNA function. However, RNAi-based therapeutics often faces *in vivo* instability because naked nucleic acids are subject to enzyme degradation before reaching the target sites. Therefore, an effective, safe and stable bio-responsive delivery system is necessary to protect the nucleic acids from serum degradation and assist their entrance to the cells. Since neuronal cells are non-regenerating, to design engineered miRNAs to be delivered to the central nervous system (CNS) for long term gene expression and knockdown is representing an enormous challenge for scientists. This article provides an insight summary on some of the innovative strategies employed to deliver miRNA into target cells. These viral and non-viral carrier systems hold promise in RNA therapy delivery for neurodegenerative diseases.

## Introduction

miRNAs play important roles in modulating gene expression in varying cellular processes and disease pathogenesis. miRNAs are small endogenous non-coding RNAs that can regulate gene expression by entering the RNAi pathway through interacting with complementary mRNAs at the post-transcriptional level by base-pairing and repress translation of target mRNAs. Specifically, they act as sequence-targeting guides which associate with the RNA-induced silencing complex (RISC) to knockdown the mRNAs (Fiore et al., 2011). Since the first miRNA replacement therapy entering phase 1 clinical trial in ClinicalTrials.gov (2013b), miRNAs have been extensively studied as a promising therapeutic drug class with proved extraordinary results in cancers and many other diseases, including neurodegenerative diseases. For example, miR-145 is recognized as a tumor-suppressor in several types of cancers including lung, prostate, gastric and breast cancers (Hu et al., 2016; Takahara et al., 2016; Tang et al., 2016; Zhang Y. et al., 2016). Viral infection also strongly influences the quantity and distribution of miRNAs within the host cells (Sharma and Singh, 2016). miR-612, miR-3654 and miR-3651 were reported to actively involve immune responses in peripheral blood of myasthenia gravis patients (Barzago et al., 2016). The ongoing miRNA-based vaccine researches aiming to put an end to the spread of Ebola virus infection was another example (Golkar et al., 2016). Numerous studies have demonstrated the potential of using miRNA-targeted therapy in improving hyperlipidemia, atherosclerosis and angiogenesis (Lovren et al., 2012; Lippi et al., 2013; Lima et al., 2016). Furthermore, there were growing evidences showing their crucial involvement in regulating a variety of pathogenic mechanisms in multiple sclerosis, leukodystrophies and perinatal hypoxia-ischemia (Galloway and Moore, 2016).

## miRNA as Therapeutics in Neurodegenerative Disease

It is estimated that 70% of known miRNAs are expressed in the brain and control brain development by regulating essential signaling pathways required in synaptogenesis, neuronal plasticity, neurite outgrowth and memory processes (Nadim et al., 2016). Several miRNAs are identified as important biomarkers in the pathogenesis of motor and neuro-cognitive diseases such as Alzheimer’s (AD), Huntington’s and Parkinson’s diseases (PD) and amyotrophic lateral sclerosis. For instance, a study of expression profiling of 328 miRNAs in the anterior temporal cortex in AD patients showed evidence of decreased expression in 13 miRNAs (Hébert et al., 2008). Other important miRNAs, including miR-153 and miR-339-5p were found significantly lowered in autopsied AD brain tissues (Long et al., 2012, 2014). On the other hand, over expression of miRNA 34a in the hippocampus was proved to be involved in anxiety-like behavior in patients with AD (Zhang Y. L. et al., 2016). Upregulating of miR-9, miR-25b and miR-128 but down-regulating of miR-124a in hippocampal region was also observed in AD brain samples (Hugon and Paquet, 2008). An interesting finding of miR-34c, which is required for memory function, suggests that targeting miR-34c could be a suitable approach to treat memory impairment in AD (Zovoilis et al., 2011). Overexpression of miR-182 in lateral amygdala was also reported to disrupt long-term amygdala-dependent memory formation (Griggs et al., 2013).

The abundant miR-133b in the mid-brain of PD patients was the first miRNA reported to be deficient in dopamine neurons (Kim et al., 2007). The decreased expression of miR-34b and miR-34c in the brain was found to associate with cell death with altered mitochondrial activity and oxidative stress in PD (Junn and Mouradian, 2012). Furthermore, loss of miR-155 occurred in correspondence to reduce pro-inflammatory responses to α-synuclein and block α-synucle in-induced neurodegeneration in PD (Thome et al., 2016). The role of miRNA-125b was also correlated as a key mediator in sustaining inflammatory signaling in microglia (Parisi et al., 2016).

Despite the fact that miRNAs are important regulators in modulating brain functions in neurodegenerative diseases, it is very challenging to deliver these miRNAs into the central nervous system (CNS) because the blood–brain barrier (BBB) hinders the accumulation of active compounds in the CNS, thus limits their transfection efficiency. To modulate these miRNA activities, there are currently two strategies involved; first one is to therapeutically restore the suppressed miRNA level by miRNA mimics (agonist), and the other one is to inhibit miRNA function by using anti-miR (antagonist) to repress overactive miRNA function (van Rooij and Kauppinen, 2014). In general, RNAi-based therapeutics often has a problem of poor stability because naked nucleic acids are subject to enzyme degradation before reaching the target sites. For that reason, an effective, safe and stable bio-responsive delivery system is necessary to protect the nucleic acids from serum degradation and assist their entrance into the cells (Chen et al., 2015). To efficiently deliver miRNAs into the body system in a safe and controlled manner without losing the payload before entering the cell targets still remains an ultimate challenge today. *In vivo* delivery of miRNA mimics may also generate considerable risks when they are taken by non-target tissues that do not express the miRNA of interest, resulting in potential off-target unwanted side effects.

The oligonucleotide-based therapy in neurodegenerative diseases that entered clinical trials was published in 2014. The delivery platform in these trials was mainly naked ASO delivery without vectors (Magen and Hornstein, 2014). Since that time, no significant new developments have been done. A summary of new miRNA studies entering clinical trials in the past 2 years was presented in Table 1. None of them was miRNA therapeutics.

**Table 1:** **Clinical trials studied miRNA and neurodegenerative diseases that were registered in 2014–2016**.

Condition	Study type	Study design	Studied RNA	ClinicalTrials.gov Identifier	Sponsor country	Ref.
Alzheimer’s disease	Observational	Cross-sectional case control	miRNA 107	NCT01819545	China	2013a
Amyotrophic lateral sclerosis	Observational	Prospective cohort	miRNA expression	NCT01992029	France	2013c
Mild cognitive impairment Alzheimer disease, Parkinson disease	Intervention	Behavioral: 3 months exercise intervention program	Role for myokines and miRNAs	NCT02253732	Slovakia	2014
Glioma, neurofibromatosis type 1	Observational	Prospective case-only	miRNA expression patterns	NCT01595139	USA	2012

The following sections provide an insight summary on some of the innovative strategies used to deliver miRNA into target cells, mostly in cancers. However, these research results could be inspired for the application of many miRNA therapeutic strategies for neurodegenerative diseases.

## Vector Systems to Deliver miRNAs

### Viral Vector Systems

One of the older strategies was to use viruses as vectors for miRNA delivery into neurons of the mammalian brain. The selection of the appropriate serotype of vector depended on numerous factors like target specific tropism, efficient transgene expression and the ability to cross the BBB. Many viruses have been exposed for this purpose with promising transfection efficiencies in the past. For example, recombinant adeno-associated virus (rAAV) was used as mediators for delivery miRNA134 in mice resulting in almost 100% transduction efficiency (Christensen et al., 2010). rAAV can target non-dividing cells and show transgene expression in a long term with relatively non-pathogenic and considered safe nature (Beutler and Reinhardt, 2009). Clinical trials of rAAV gene therapy in PD also showed that they were well tolerated in the human CNS and expressed persistence of transgenes in a long-term (Kaplitt et al., 2007; Marks et al., 2008).

Other viral vectors such as adenovirus, retrovirus and lentivirus have also been widely researched. Compared to AAVs, adenoviruses are easy to produce and may incorporate large transgenes with a high level of expression that can activate stronger dose-dependent innate and adaptive immune responses (Nayak and Herzog, 2010). Transduction with adenovirus vectors showed more efficient expression of therapeutic genes in the liver, but a lesser extent in the lung and brain (Carnero et al., 2011).

On the other hand, the modification of lentivirus tropism has been made toward astrocytes with neuron-specific miR124 to eliminate residual expression in neuronal cells for cell type-specific gene transfer to the CNS (Colin et al., 2009). Effective transfection of recombinant human miRNA-7-3 gene into human glioma U251 cells to suppress glioma cell growth by lentiviral vectors were also reported (Dong et al., 2012). Recently, lentivirus-mediated miRNA-210 has been delivered in ischemic mouse brain and demonstrated the improvement of long-term outcomes for stroke therapy (Zeng et al., 2016).

A possible commonly held notion is that viruses are bioactive. They simply attach to a receptor on the cell surface and are easily taken into host cells. However, there are many drawbacks of using viruses as carrying vehicles, especially the safety concern due to the immunogenicity and the risk of triggering an oncogenic transformation by viruses. In addition, viral vectors initiate innate immunity and antigen specific adaptive immune responses leading to reduced efficiency of gene transfer. At high vector doses, transient innate immune responses were seen in naïve mouse brain parenchyma in one study (Lowenstein et al., 2007). Injections of additional doses to the opposite hemisphere demonstrated significantly greater immune response and lower transgene expression. In gene therapy, each vector system generates its own immunological responses; some are more active to innate immunity and others to adaptive immunity. Furthermore, pharmaceutical scale productions of high-quality and quantity viral vectors also impose another concern, leading to limit their applicability.

### Non-Viral Vector Systems

Past research evidences indicated that non-viral vehicles were stable, non-immunogenic, and easy to be modified to meet specific needs for overcoming the physicochemical and biological barriers, subsequently achieving higher transfection efficiency. Here, some of the most popular novel non-viral gene vectors were reviewed, yet others were also available throughout various literature resources.

#### Lipid-Based Carriers

##### Cationic liposomes

Liposomes are lipid-based vesicles made of amphiphilic phospholipid bilayers with an aqueous core. Research results showed that liposomes could be used as a promising tool for targeted gene delivery (Alvarez-Erviti et al., 2011; Pinzón-Daza et al., 2013). When they are made charged with cationic lipids, such as cholesterol, dioleoylphosphatidyl ethanolamine, phosphatidylcholine and unsaturated fatty acids, they are able to form ion pairs with anionic phospholipids on the endosomal membrane, thus promoting the release of encapsulated miRNAs after endocytosis. A typical cationic liposome usually comprises of cationic lipids, neutral lipids and Polyethylneglycol (PEG)-lipids. Neutral lipids, also called “helper lipids” which are used to increase the stability and decrease the toxicity of the cationic lipids (Wang et al., 2013). For example, a cationic lipoplexes-based carrier system for miR-29b delivery was reported to be able to suppress tumorigenicity by restoration of miR-29b in non-small–cell lung cancer. These lipids contained a cationic lipid, 1, 2-di-*O*-octadecenyl-3-trimethylammonium propane, a neutral lipid, cholesterol and d-α-Tocopheryl PEG 1000 succinate which was formulated as empty liposome used to entrap the therapeutic agent miR-29b.

The positively charged lipoplexes proved to facilitate the interaction with the negatively charged cell membrane, providing more efficient cellular uptake to target multiple oncogenes in non-small–cell lung cancer A549 cells (Wu et al., 2013). Similarly, a cationic liposome vehicle composed of a cationic lipid 2-dioleyloxy-N,N-dimethyl-3-aminopropane, egg phosphatidylcholine and cholesterol was developed for systemic delivery of exogenous miR-122 mimic in hepatocellular carcinoma therapy, resulting in a significant inhibition of expression of miR-122 target genes. PEG was attached on the surface of the Lipid nanoparticle (LNP) to increase the *in vivo* stability and circulation half-life time (Hsu et al., 2013).

Another interesting study showed the development of bubble liposomes to be used as a diagnostic and therapeutic system. Bubble liposomes contained a cationic lipid, 1,2-distearoyl-3-dimethylammonium-propane that entrapped ultrasound echo-contrast gas inside the core vesicle. miRNAs were then loaded onto the surface of these bubble liposomes. The results suggested that miRNA-bubble liposomes could be detected by diagnostic ultrasound at an ischemic target site. And miR-126 was delivered following therapeutic ultrasound, leading to the induction of angiogenic factors and improved blood flow in angiogenic treatment. The combination of ultrasound and bubble liposomes systemic delivery was proved to be a useful tool for ultrasound imaging and miRNA delivery (Endo-Takahashi et al., 2014).

##### Cationic lipid nanoparticles (LNP)

A targeted delivery of miR125a-5p via LNP platform for the treatment of HER2 positive metastatic breast cancer was investigated. First, a lipid solution mixture composed of L-phosphatidylcholine, 1,2-dipalmitoyl-sn-glycero-3-phopshoethanolamine and cholesterol was mechanically extruded to create unilamellar vesicles of LNP, followed by conjugation of Hyaluronic acid (HA) on the surface of the LNP. The formulation was finalized by lyophilization. To entrap the miRNA, lyophilized lipid powder was rehydrated with FITC-Dextran tagged human miR125a-5p mimic solution. A mono-dispersed population of nanoparticles (NPs) was obtained using this method of preparation (Hayward et al., 2016). This type of fabrication might be tested for miRNAs of interests for neurodegenerative diseases.

#### Gold Nanoparticles (AuNPs)

Gold Nanoparticles (AuNPs) have shape-dependent optical and electronic features, high affinity for biomolecules and are feasible for surface modification. One study indicated that by surface modification with thiolated RNAs, these AuNPs could be used to deliver hsa-miR-145 to facilitate the delivery of miRNA into prostate/breast cancer cells (Ekin et al., 2014). Another layer-by-layer technique was also described using alternating charged polyelectrolytes to prepare multilayered siRNA coated AuNPs. In this method, poly-L-lysine (PLL), a positively charged polyelectrolyte, was coated onto the surface of AuNPs first. Then siRNA was added and conjugated on the surface of the PLL-AuNPs. Up to four layers of PLL and three layers of siRNA were coated by this assembly approach. The results showed that siRNA was released gradually and achieved more than 80% gene silencing effect which was found to correlate with the number of siRNA layers (Lee et al., 2011). These techniques could be also applied for CNS miRNAs.

#### Cationic Polymer-Based Carriers

##### Bioresponsive hyperbranched polymers

Polycation-based NP delivery systems were shown in many studies to be able to improve both extracellular and intracellular delivery of RNAi-based drugs. A bioresponsive hyperbranched polymer delivery system response to the cytoplasmic redox conditions was reported. The cationic poly(amido amine; PAMAM) hyperbranched polymers containing reducible and non-reducible linkage units were synthesized to form inter-polyelectrolyte polyplexes with miRNA which exhibited redox-activated disassembly in response to the redox potential gradient between the extracellular and intracellular environment. This system could facilitate endosomal rupture, triggered release and maximum intracellular target interaction for gene silencing (Rahbek et al., 2010).

##### Hyaluronic acid-chitosan nanoparticles

HA is a natural anionic polysaccharide, which can be recognized by cells and improve cellular uptake through HA receptor-mediated endocytosis. A novel approach that can simultaneously deliver miR-34a and doxorubicin into HA-chitosan NPs against triple negative breast cancer was studied. Anionic HA and cationic chitosan were used as the driving forces to encapsulate negatively charged miR-34a and positively charged doxorubicin through a cross-linker tripolyphosphate (Deng et al., 2014).

##### Poly(lactic acid-co-glycolic) acid (PLGA) nanoparticles

miR-124 is known to associate with various brain pathologies and neurodegenerative disorders. Significant decrease of miR-124 is usually found in PD (Gong et al., 2016); therefore, by increasing miR-124 intracellular levels to promote neurogenesis of neural stem cells in sub-ventricular zone of the brain through induction of miR-124 regulated neuronal differentiation might improve functional outcome in PD. A study used biocompatible and traceable Poly(lactic acid-co-glycolic acid; PLGA) NPs containing perfluoro-1,5-crown ether that can be tracked by ^19^F-MRI. Protamine sulfate was then surface conjugated to complex miR-124 to enhance the brain repair in PD mice models. The results showed remarkable decreased expression of Sox9 and Jagged1, two miR-124 targets and stemness-related genes. Use of miR-124-PLGA NPs demonstrated a new theranostic approach for neurodegenerative diseases (Saraiva et al., 2016).

##### Hydrogels

Hydrogels have porous hydrophilic networks that are able to take up large amounts of fluids while maintaining their semisolid morphology (El-Sherbiny and Yacoub, 2013). The high water content and soft nature of hydrogels promote faster drug release from the gel matrix. A combination of a miRNA mimic and an antagomiR together with another miRNA, which was a complement of the replacement strand, was assembled into triple-helix structures in one study. These triple helices were then conjugated to dendrimers and mixed with dextran aldehyde to form an adhesive hydrogel. This nano-hydrogel containing therapeutic miRs could be injected directly onto the surface of tumors with high efficiency (Conde et al., 2016).

##### Polyethylenimines nanoplexes

Polyethylenimines (PEIs) are highly positively charged polymers that are able to form nano-complexes with small RNAs to achieve RNA protection and improve intracellular release by the “proton sponge effect”, leading to increased membrane permeability (Höbel and Aigner, 2013). However, non-modified PEI may cause cytotoxicity due to high charge density. Thus modification of PEI might improve the physicochemical properties of PEI. A study of reducible PEI was reported by synthesis of high molecular weight PEI (25,000 Da) with cetyl bromide, and then conjugated with PLGA polymer and cross-linked with HA to facilitate the cellular uptake of tumor-suppressor miR-145 (Liang et al., 2016).

Another study used ionized polysaccharide from a Chinese medicine *Angelica sinensis* by chemical modification of a branched low molecular weight PEI (1200 Da) to form NPs for loading plasmid DNA encoding transforming growth factor beta 1 into mesenchymal stem cells. The results showed a greater transfection efficiency and less toxic comparing to Lipofectamine 2000 and PEI (25 kDa; Deng et al., 2013).

#### Carbon-Based Carriers

Another novel nanocarrier system was formed by self-assembled protamine sulfate-functionalized nanodiamonds for miRNA-203 delivery in esophageal cancer (Cao et al., 2013). Nanodiamonds are carbon-based NPs with stable inert core sizes of 2–8 nm. They are non-toxic, highly biocompatible and are capable of loading peptides, small molecules, antibodies, DNAs and RNAs with high efficiency and low toxicity (Zhao et al., 2016). However, unmodified nanodiamonds normally tend to agglomerate and precipitate in solution. In this study, protamine sulfate was first adsorbed on the surface of nanodiamond NPs to create positively charged NPs. Then negatively charged miR-203 mimics was attached to the positively charged surface of nanodiamond NPs through electrostatic interactions. The results showed a higher transfection efficiency obtained by this system when compared with the “gold standard” PEI 25K.

Another strategy was to use polymer functionalized carbon nanotubes (CNTs) as effective means to regulate target gene expression and angiogenesis. CNTs were coated with two different polymers, PEI or PAMAM, to improve the electrostatic interaction of CNTs with the negatively charged siRNAs or plasmid DNA, followed by conjugation of miR-503. The results showed an increasing nucleic acids loading and improving cell uptake (Masotti et al., 2016).

#### Mesoporous Silica Nanoparticles

A smart system based on mesoporous silica NPs capable of simultaneous delivery and controlled release of anti-miRs and small molecule into target cells was developed. Mesoporous silica NPs possessed advantages of high surface areas, large loading capacity, chemically modifiable surfaces and low toxicity. This system was intended to be stimuli-responsive to the amounts of the expression levels of endogenous miR-122 in hepatocellular carcinoma cells (Yu et al., 2015).

#### Magnetic Nanoparticles

An iron oxide nanocomposite NPs, termed magnetic reagent for efficient transfection (MagRET) comprised of a maghemite core that is surface treated with lanthanide Ce^3/4+^ cations was fabricated as gene carrier. PEI was then attached to this maghemite core to form an antisense miRNAs NP complex for silencing several types of RNAs, including miRNAs (Lellouche et al., 2015). Another system contained a combination of a cationic chondroitin sulfate conjugated with PEI and superparamagnetic iron oxide NPs (SPION) for magnetofection in glioblastoma multiform therapy. Chondroitin sulfate has the capacity of targeting CD44 which enhances its crossing BBB. It has been reported not only gliomas express the CD44 but also brain micro vascular endothelial cells do. An external magnetic field was applied to guide the therapeutic genes in the form of magnetoplexes to the surface of target cells for more efficient plasmid DNA delivery (Lo et al., 2015).

#### Cationic Dendrimers

PAMAM was the first dendritic molecule used as gene carrier. The surface of the PAMAM dendrimer is highly cationic charged, resulting in electrostatic interaction with the negatively charged nucleic acid. The dendrimer/nucleic acid nanocomplexes can be further conjugated with targeting ligands such as transferrin and lactoferrin for the delivery of plasmid DNA into the brain with a high brain penetration capability of targeting BBB (Dehshahri and Sadeghpour, 2015).

## Conclusion

Current available data indicated significant regulatory roles of miRNAs in the pathogenesis of neurodegeneration. These remarkable miRNAs could be used as diagnostic markers and therapeutics for many progressive neurodegenerative diseases. However, more researches should be performed on the pharmacokinetics of miRNA in the body to understand the threshold copies of miRNA that should be replaced or repressed in each disease state. To design specific miRNAs carriers for long term gene expression and knockdown in CNS is representing an enormous challenge for scientists to overcome.

## Author Contributions

The author confirms being the sole contributor of this work and approved it for publication.

## Conflict of Interest Statement

The author declares that the research was conducted in the absence of any commercial or financial relationships that could be construed as a potential conflict of interest.

## Abbreviations

AD: Alzheimer disease
AuNP: Gold nanoparticle
BBB: Blood–brain barrier
CNS: Central nervous system
CNT: Carbon nanotubes
HA: Hyaluronic acid
LNP: Lipid nanoparticle
NP: Nanoparticle
PAMAM: Poly(amido amine)
PD: Parkinson’s disease
PEG: Polyethylneglycol
PEI: Polyethylenimines
PLGA: Poly(lactic acid-co-glycolic acid)
PLL: Poly-L-lysine
rAAV: Recombinant adeno-associated virus.
